# Nanocellulose-Based Films for Surface Protection of Wooden Artefacts †

**DOI:** 10.3390/ijms252413333

**Published:** 2024-12-12

**Authors:** Paulina Kryg, Bartłomiej Mazela, Waldemar Perdoch, Mariusz Jancelewicz, Magdalena Broda

**Affiliations:** 1 Department of Wood Science and Thermal Techniques, Faculty of Forestry and Wood Technology, Poznan University of Life Sciences, ul. Wojska Polskiego 38/42, 60-637 Poznan, Poland; p.jarmuszkiewicz@wp.pl; 2 Faculty of Forestry and Wood Technology, Poznan University of Life Sciences, Wojska Polskiego 28, 60-637 Poznan, Poland; bartlomiej.mazela@up.poznan.pl (B.M.); waldemar.perdoch@up.poznan.pl (W.P.); 3 NanoBioMedical Centre, Adam Mickiewicz University, Wszechnicy Piastowskiej 3, 61-614 Poznan, Poland; mariusz.jancelewicz@amu.edu.pl

**Keywords:** nanocellulose films, silane modification, wood consolidation, surface treatment, wooden cultural heritage

## Abstract

This research investigated the selected properties of nanocellulose films intended to serve as protective patches on fissured surfaces of wooden artefacts. The effects of their plasticisation with glycerol and functionalisation with selected silanes ((3-Glycidyloxypropyl)trimethoxysilane, and Methyltrimethoxysilane) were also determined. The obtained pure cellulose nanopapers (CNPs) had a homogeneous and compact structure but were very brittle, stiff, and wavy. Functionalisation with silanes made their structure more packed and reduced their equilibrium moisture content by 87–96%, depending on the type and concentration of the silane. Silane functionalisation also slightly improved nanopapers’ resistance to moulds. Plasticisation with glycerol provided CNPs with higher flexibility and resistance to fracture and made them flatter and smoother, reducing the wettability of their surfaces but increasing their hygroscopicity (EMC values increased 1.7–3.5 times for pure CNPs and 5–33 times for functionalised CNPs) and vulnerability to mould infestation. All prepared nanopapers can be easily glued to the wood surface and colour-matched using a nitro wood stain, oil paint or waterborne acrylic paint. The research showed that cellulose nanopapers modified with silanes and plasticised with glycerol seem to be a promising solution for protecting the cracked surface of wooden artefacts against further degradation due to external conditions.

## 1. Introduction

Nanocellulose (NC) is a highly versatile, natural, renewable, and sustainable nanomaterial with several useful properties, including low density, high surface area, good thermal stability, high tensile strength and Young’s modulus, good insulation, low gas permeability, optical transparency and overprint ability. All these make it suitable for multiple industrial purposes, including biomedical applications, food packaging, wastewater treatment, electronics, cosmetics and textile production [1,2,3].

Nanocellulose is a form of cellulose—a structural polysaccharide of plant, tunicate and several algae tissues—with at least one dimension sized between 1 and 100 nanometres. Chemically, it is a linear, semicrystalline polysaccharide composed of repeated D-glucopyranose units linked by β-(1-4)-glycosidic bonds. Each unit contains three hydroxyl groups, with a hydrogen atom acting as an electron donor and adjacent oxygen acting as an electron acceptor. As a result, each hydroxy group can react with many other molecules, forming various derivatives [4,5,6]. Moreover, through hydrogen bonds, individual nanocellulose molecules can interact with one another, forming firm, extensive, self-assembled structures with excellent film-forming and barrier properties [7,8]. Depending on the production method, four types of nanocellulose are possible: cellulose nanocrystals (CNCs), cellulose nanofibers (CNFs), regenerated nanocellulose (RNC), and bacterial nanocellulose (BNC). They differ in structure and properties, which affects their applications [9,10,11,12,13,14].

Among the nanocellulose features that have recently been widely explored are their excellent film-forming properties, which enable the production of nanopapers with high strength and barrier properties [15,16,17].

Nanopaper (nanocellulose film, cellulose nanopaper, CNP) is a biodegradable, non-woven, 2D flat and foldable film composed mainly of nanocellulose particles. It can be manufactured using different techniques, including casting, coating, and filtration. Nanopaper differs from traditional paper in fibre size and the properties resulting from high adhesion between nanofibrils due to the formation of multiple hydrogen bonds. Consequently, superior mechanical characteristics (high modulus of elasticity and tensile strength) and the ability to form compact, homogenous structures with morphological defects distributed more regularly than in paper composed of larger fibres can be observed [18]. In addition, CNP has low weight, good barrier properties, high thermal stability, low thermal expansion coefficient, controllable optical properties, sustainability, and low environmental impact. However, due to its hydrophilic character, although mechanically strong under dry conditions, CNP is easily weakened when humidity increases, significantly limiting its applications. Combining NC with other nanoparticles or further functionalisation of its molecules with different chemicals can reduce its hydrophilicity and additionally enrich NP with ancillary functionalities such as fire retardancy, magnetic properties, and electrical conductivity, tailored to the needs of various traditional and higher-end products, making it a great alternative to the conventional petroleum-based polymers used so far [15,16,19].

Conservation of wooden cultural heritage exposed outdoors is one of the areas that require new materials for effective preservation of unique artefacts for future generations. In particular, filling voids and cracks in wooden artefacts constantly exposed to changing weather conditions seems to be exceptionally important to keep the integrity and aesthetic of a historical wooden object. It is a complex issue burdened with the danger of causing further unintentional degradation of the original wood piece. Although various substances have been used for this purpose, no ideal solution has been developed so far that would meet all the requirements for a filler, such as short and long-term chemical and physical stability, mechanical strength similar to wood, low shrinkage/swelling in response to the temperature and humidity changes, good adhesion to wood, high resistance to biodegradation, reversibility, non-toxicity, optical matching and causing no aesthetic changes [20,21,22,23,24,25]. An improperly selected filler may cause progressive chemical and mechanical damage to the artefact, promote its further fungal, microbial, algae or insect infestations, and, as a result, lead to the loss of its integrity, aesthetics and historical values and even to complete disintegration [22,26].

Apart from developing new materials for gap filling [23], a new idea emerged of securing the cracks and voids in historical wood with external “patches” made of NPs. Transparent nanocellulose films provide excellent gas barrier properties and great stability at various light, temperature, and humidity levels, and they remain stable with age. The optical properties of nanopapers also remain unchanged over time [27,28,29]. Higher nanocellulose concentrations in CNP increase their stiffness and improve tensile properties at the breaking point [30]. The performance of thin nanocellulose films may be enhanced using glycerol as a plasticiser, which reduces their brittleness, increases flexibility, and improves the oxygen barrier properties of CNPs under dry conditions [31,32,33]. Adding glycerol up to 30% of dry matter can also decrease the moisture diffusion coefficient and porosity of pure nanocellulose films [34,35,36]. Further silane treatment can effectively improve interfacial adhesion and provide super-hydrophobic properties to a nanopaper surface [6,37,38], increasing their thermo-oxidative stability, UV resistance, and mechanical properties through cross-linking reactions [39,40].

Patches with nanocellulose films can be applied directly onto a wooden substrate or indirectly using chemical pre-modification and can be easily further functionalised to engineer smooth, thin interfaces with specific properties [41]. Considering the abovementioned properties and advantages, suitably functionalised CNPs glued to wood’s surface seem to be promising novel materials for protecting cavities and cracks in historical wooden objects against harmful, changeable weather conditions and further biological, chemical and mechanical degradation. An undeniable advantage of using nanocellulose-based films is the reversibility of the conservation process, which is important from the perspective of conservation ethics [42].

This study aimed to prepare cellulose nanopapers intended to serve as innovative protective patches for cracks and voids in wood surfaces against water, dust, and dirt penetration and, consequently, microorganism infestation. To achieve this, nanopapers were produced, and the effect of glycerol and silane treatment on their performance, including film-forming, moisture and antifungal properties, was investigated.

## 2. Results and Discussion

### 2.1. Composition, Morphology and Selected Properties of Nanopapers

The overall composition and selected properties of prepared pure nanopapers are presented in Table 1. The abbreviation scheme for the samples is as follows: for untreated samples, we use CgY, where C represents cellulose nanocrystals, g is glycerol and Y is the CNC to H_2_O ratio, where A is 1:1.5 and B is 1:4, while for silane-treated samples, we use XgY, where X can be G, which represents nanocellulose functionalised with (3-Glycidyloxypropyl)trimethoxysilane, or M, which represents nanocellulose functionalised with Methyltrimethoxysilane; g is glycerol; Y is the silane concentration, where 3 represents 3%, 10 is 10% and 30 is 30%.

**Figure 1:**
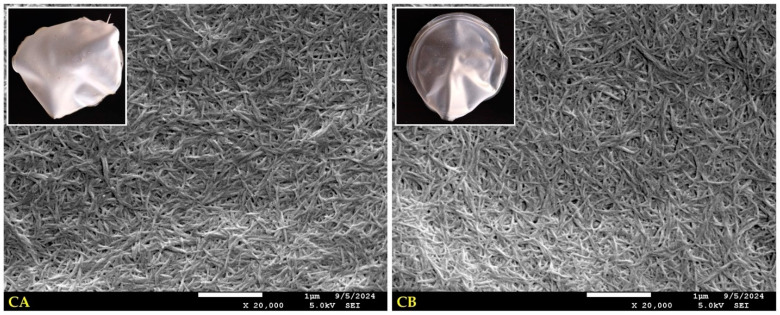
SEM images and macroscopic pictures of unmodified nanocellulose papers: CA—CNC:H_2_O ratio 1:1.5; CB—CNC:H_2_O ratio 1:4.

Although the amount of CNC did not affect any of the evaluated properties of pure nanopapers regarding their flatness, brittleness and removal from the pad, the macroscopic picture of two variants was different (Figure 1). Depending on the water content during the nanopaper production stage, CA and CB samples scatter and reflect light differently. As a result, the CB sample seems more transparent than the CA sample. The influence of water amount during nanopaper production on their structure is relatively easy to observe in SEM images. Increasing the amount of water most likely significantly changed the interactions between hydrogen bonds in CNC suspension. Suspending CNC in water (ratio 1:4) during the formation of nanopapers probably caused the particles and cellulose nanoparticles to move away from each other, disturbing the original energy balance between the hydroxyl groups contained in cellulose. Consequently, during water evaporation, new and more stable structure was created for the CB samples compared to the CA nanopapers.

**Figure 2:**
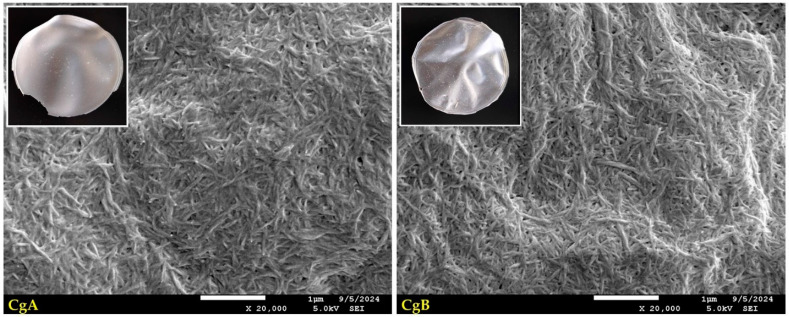
Macroscopic pictures and SEM images of nanocellulose papers plasticised with glycerol: CgA—CNC:H_2_O ratio 1:1.5; CgB—CNC:H_2_O ratio 1:4.

Regardless of CNC concentration, adding glycerol improved the flexibility (from 1 to 4) and flatness (from 2 to 4) of CNPs significantly without affecting the easiness of their removal from pads (Table 1). Similar results were obtained for nanopapers made of both 1:1.5 and 1:4 CNC concentrations. Macroscopic pictures and SEM images of dry pure nanopapers with and without glycerol are presented in Figure 2. Glycerol increased the CNPs’ flexibility and resistance to fracture. The SEM image quality of nanopapers with glycerol addition was slightly worse than the SEM images of nanopapers without glycerol. The probable reason for this phenomenon was that the plasticiser increased the elasticity but reduced the number of hydrogen bonds in the cellulose network, and the applied electrons from the SEM quickly destroyed the nanopaper structure. Consequently, the samples gradually degraded during imaging, and the image quality decreased. The lower ratio was selected for further experiments to prepare functionalised CNPs to keep an equal amount of CNC in pure and modified nanopapers.

Analogical properties were evaluated for functionalised CNPs (Table 2). In general, for both silanes used, along with their increasing concentration, the increase or no change in the quality of evaluated individual properties of nanopapers was observed, resulting in a rise in the final average scores obtained. The only exception was the G30 sample, for which the increase in the (3-Glycidyloxypropyl)trimethoxysilane (GOPTMOS) concentration was 30%. This sample showed improved flexibility and flatness, but also hindered the removal of the nanopaper from the pad. More remarkable improvement in the analysed properties was observed for GOPTMOS-treated nanopapers compared to those treated with Methyltrimethoxysilane (MTMOS), presumably due to its higher reactivity and the ability for cross-linking through not only alkoxy groups also present in MTMOS but also through the glycidyl group, which improves the film’s elasticity and tensile strength [39].

The addition of glycerol to the CNC suspension modified with 3% GOPTMOS significantly improved the quality of the CNP; the obtained nanopaper was perfectly flat, smooth and flexible, but its removal from the pad was difficult due to its high stickiness (nanopaper stuck to the pad’s surface). In the case of variants with higher concentrations of GOPTMOS (10% and 30%), it was impossible to produce nanopapers with the addition of glycerol because the obtained suspensions remained as a dense semi-liquid substance, sticky and oily even after 2 weeks of drying at room temperature. Glycerol also improved the properties of MTMOS-modified CNPs. Nanopapers without glycerol were generally characterised by high brittleness with a ragged, corrugated surface, while their glycerol-modified alternatives demonstrated much higher flexibility and flatness. Even though the presence of glycerol hindered the removal of the 3% MTMOS variant from the pad and caused some waviness of the 10% MTMOS-treated CNP, the average scores for each MTMOS-treated variant were better compared to MTMOS-treated CNPs without glycerol.

**Figure 3:**
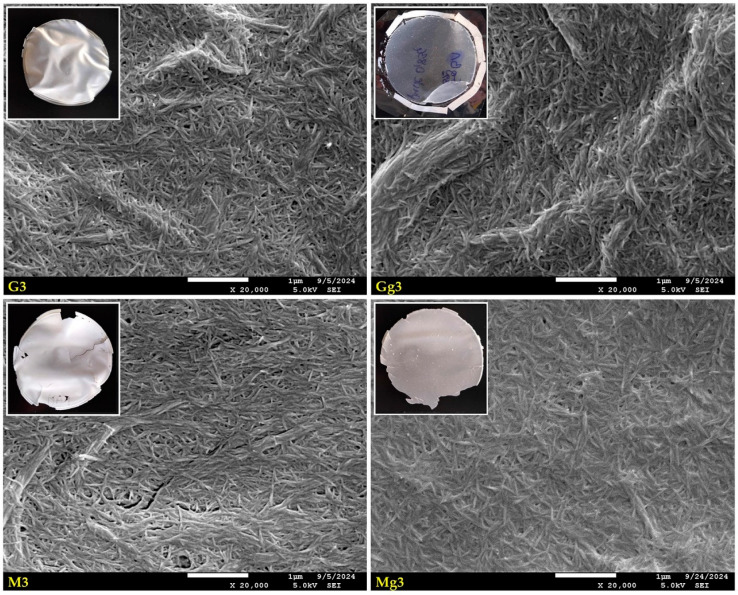
Macroscopic pictures and SEM images of nanocellulose papers functionalised with silanes: G3—modified with 3% of (3-Glycidyloxypropyl)trimethoxysilane; M3—modified with 3% of Methyltrimethoxysialne, and their plasticised equivalents Gg3 and Mg3.

SEM imaging (Figure 3) revealed the surface morphology of modified nanocellulose papers. Under ×20,000 magnification, a dense, uniform network of individual nanocellulose whiskers was visible. It seems similar for unmodified NPs (Figure 1) and paper modified with GOPTMOS (Figure 3). CNC modified with MTMOS significantly changes the structure of nanopapers. In the SEM pictures, more horizontal than vertical distribution of cellulose chains was quickly recognised. The observed results show a strong interaction between silane and cellulose. According to the literature, the mechanism by which MTMOS changes cellulose involves the reactivity of its methoxyl groups with hydroxyl groups on the cellulose surface. This way, new siloxane bonds are formed, effectively replacing some hydroxyl groups with alkoxysilane groups in acidic or neutral-catalysed conditions [43]. As a result, the surface energy of cellulose is altered, leading to decreased wettability and enhanced hydrophobic characteristics [44]. Moreover, the structural modification induced by MTMOS can also influence the mechanical properties of cellulose-based materials. Introducing silane groups can enhance the cross-linking between cellulose fibres and change the mechanical properties and stability of the resulting materials [45]. The effect of more horizontal cellulose chain distribution was reduced when MTMOS functionalisation was run in the presence of glycerol. Changing the silane concentration did not change the structure of nanopapers significantly, as can be seen in SEM images (Figure 3).

**Figure 4:**
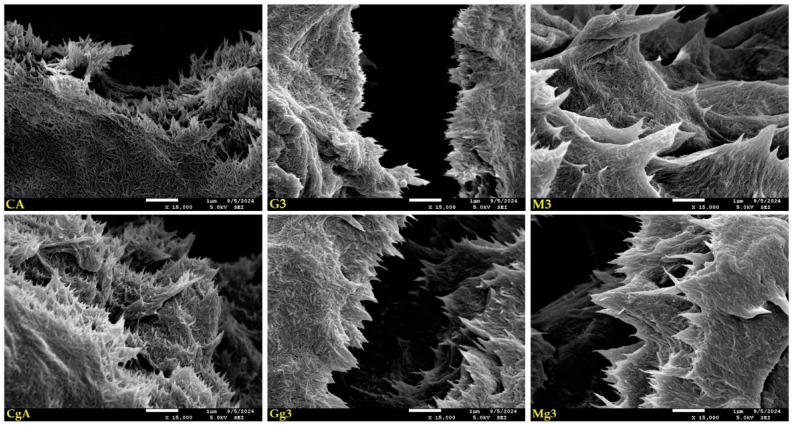
SEM images of selected unmodified and modified nanocellulose papers in destructed region: CA—unmodified with CNC:H_2_O ratio 1:1.5; G3—modified with 3% of (3-Glycidyloxypropyl)trimethoxysilane; M3—modified with 3% of Methyltrimethoxysialne, and their plasticised equivalents CgA, Gg3, Mg3.

The structural properties of nanopapers influenced by additives (silane and/or glycerol) can also be easily observed in SEM images of the destructed samples region (Figure 4). Images showing a place of tear on CNPs reveal parts of their cross-sections and internal structures. The margins of all nanopapers are serrated, but the shape of serrations differs between variants. The sharpest serrations made of smaller amounts of whiskers can be observed for unmodified CNPs (CA and CgA in Figure 4), where the glycerol-containing variant seems to have more dense wefts and individual whiskers closely glued together without empty spaces left between them. Medium-sharp serrations with more compact structures can be seen for GOPTMOS-functionalised nanopapers (G3 and Gg3 in Figure 4), with the glycerol-containing sample having less frayed edges with whiskers glued together more tightly. For MTMOS-functionalised variants (M3 and Mg3 in Figure 4), the serrations are sharp and compact, made of multiple whiskers tightly glued together, seeming to be immersed/covered with a dense liquid for both pure and glycerol-containing variants. The observed differences may indicate different mechanical parameters of the prepared nanopapers depending on their composition, and further research will be conducted to recognise them.

**Figure 5:**
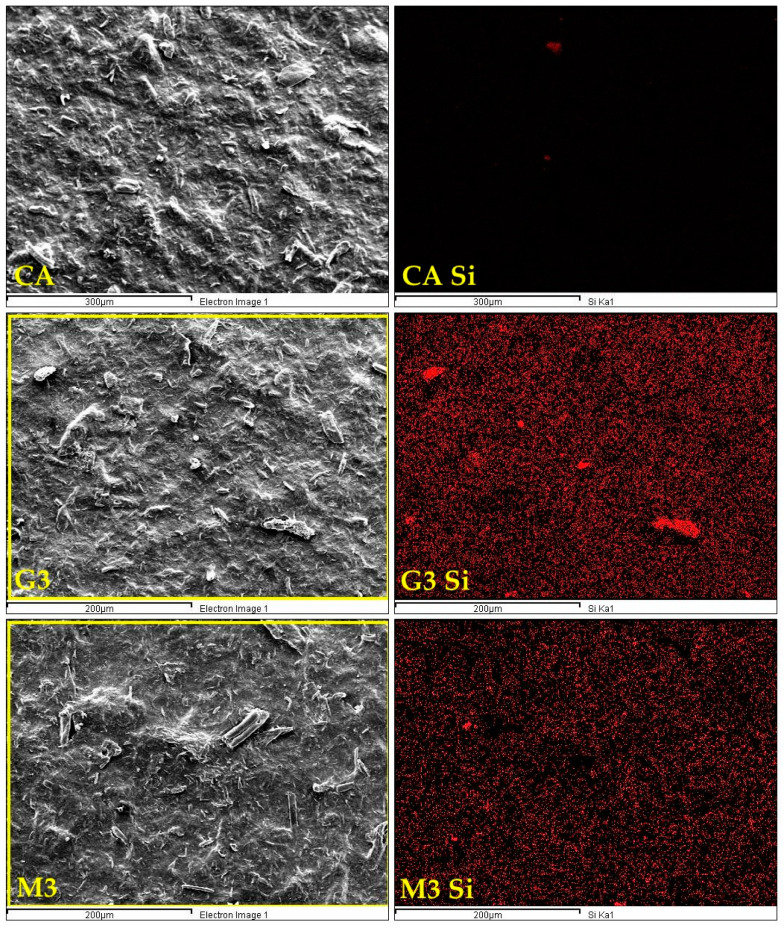
SEM EDX images of selected unmodified and modified nanocellulose papers, including electron image of the sample (on the **left**) and Si map of the corresponding area (on the **right**): CA—unmodified with CNC:H_2_O ratio 1:1.5; G3—modified with 3% of (3-Glycidyloxypropyl)trimethoxysilane; M3—modified with 3% of Methyltrimethoxysialne.

As seen in sample SEM EDX Si maps of nanopapers in Figure 5, the pure CNP sample (CA) does not contain silicon atoms, while the functionalised G3 and M3 CNPs contain silicon atoms uniformly dispersed throughout their surfaces, with their higher concentrations present in places where bigger clusters of nanocellulose whiskers occur, which suggests that silane molecules interact directly with nanocellulose particles.

### 2.2. The Water Contact Angle for Nanopapers

**Figure 6:**
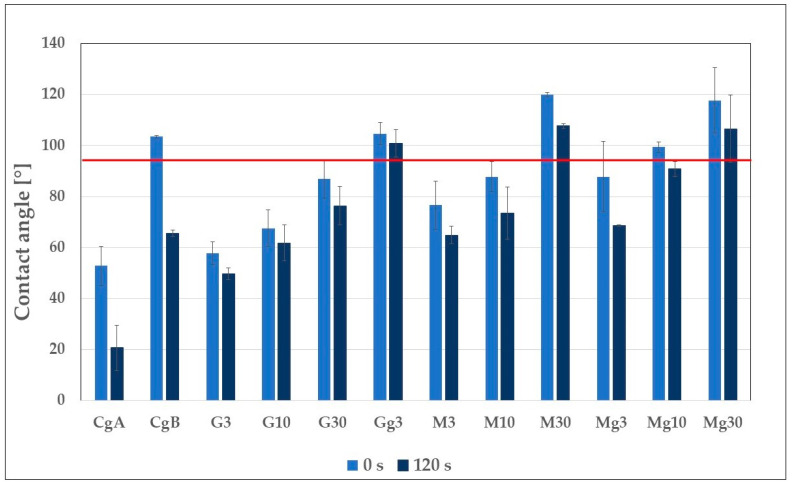
Average values for water contact angle measured for pure and functionalised nanopapers at two different times of droplet deposition: 0 s and 120 s. The abbreviation scheme is as follows: XgY, where X is as follows: C—clear non-functionalised nanocellulose, G—nanocellulose functionalised with (3-Glycidyloxypropyl)trimethoxysilane and M—nanocellulose functionalised with Methyltrimethoxysilane; g—glycerol; Y—silane concentration: 3—3%, 10—10% and 30—30%; red line at 90° divides the graph area into two parts: hydrophobic (above the line) and hydrophilic materials (below the line).

The water contact angle was measured to determine the wettability of nanopapers and assess the effect of glycerol and silane modification on CNP hygroscopicity. The graph in Figure 6 presents the average values for the water contact angle measured for pure and modified CNPs. According to the definition, a material with a water contact angle exceeding 90° is considered hydrophobic, while that with a water contact angle lower than 90° is considered hydrophilic [46]; a line at 90° was marked in the chart (Figure 6) to divide the area into two parts: hydrophobic above the line, and hydrophilic below the line.

WCA was measured only for those CNP variants that could be removed from a pad. For nanopapers based on pure nanocellulose (CA, CB), determining the wetting angle was impossible because water droplets seeped into the CNP surfaces quickly, and at the end of measurements, they were entirely absorbed. The addition of glycerol substantially changed the quality of nanopapers and enabled WCA measurements.

It is clear that the increased water ratio to CNC at the nanopaper production stage increased the material’s hydrophobic effect (CgA vs. CgB in Figure 6). Moreover, it can be observed that the standard deviation for the CgB sample was much smaller than for CgA, which may indicate its more homogeneous structure. The probable reason for the abovementioned observation was explained by SEM analysis of CA and CB differences and the influence of hydrogen bond energy changing in the presence of water (Figure 3). For both types of nanopapers, WCA was not stable over time, but it was more stable for CgB, decreasing by about 37% compared with 61% for CgA.

It is known that low-molecular-weight glycerol can easily penetrate the polymer network in films [47]. As Lavrič et al. [36] hypothesised, glycerol molecules added to the film can fill the pores between NC whiskers, thus reducing the surface porosity and increasing the surface free energy of GOPTMOS-modified nanopapers and their plasticised equivalents.

As can be seen from Figure 6, increasing concentrations of GOPTMOS had an ascending hydrophobising effect on nanopapers; the WCA of treated nanopapers raised along with increasing GOPTMOS content, reaching an average value of 76° at the end of measurement for G30, while for G3, it was only 49.7°. This was due to the chemical structure of the silane, which allowed for its reactivity with nanocellulose hydroxy groups [40,48], where the increasing concentration of GOPTMOS increased the cross-linking rate in the CNC film, reducing the number of hydrophilic groups, and thus making the material more hydrophobic [39]. Higher GOPTMOS concentrations also slightly stabilised WCA values over time; after 120 s, they decreased by about 14% for GA, while for the G10 and G30 variants, the decrease was 8% and 12%, respectively.

As mentioned above, among glycerol-treated nanopapers, only those with 3% GOPTMOS were removable from the pad and useable for further examinations. The addition of glycerol increased WCA for the Gg3 sample at the beginning of the test by about 81.5% and 102.8% at the end of the measurement compared to the non-plasticised version. It also improved WCA stabilisation over time—after 120 s, a decrease in WCA was less than 4% for Gg3, while for G3, the WCA value decreased by almost 14%. A similar effect has been reported by Lavrič et al. [36], where adding glycerol to the bacterial nanocellulose film increased the WCA value by 14% compared to unmodified film. On the one hand, it may seem surprising because glycerol is a highly hygroscopic chemical [49] which usually increases the wettability of materials where added as their component, as can be seen, e.g., in the case of chitosan-based biofilms, edible starch films or glycerol microemulsions dispersed on coal seam [50,51,52]. On the other hand, it is also known that a contact angle decreases with increasing roughness of a surface [53], and adding glycerol to various films produces smoother surfaces [54]. In our case, we hypothesise that the observed increase in WCA results from the reduction in the surface porosity and roughness by glycerol molecules filling the pores between the NC whiskers, thus increasing the surface free energy, as described by Lavrič et al. [36], while the hydrophilising effect of glycerol is neutralised by the presence of a hydrophobising silane reacting with hydroxy groups available in the CNP.

The functionalisation of nanopapers with MTMOS enhanced the hydrophobicity of their surfaces (Figure 6). Similarly to GOPTMOS-functionalised nanopapers, an increasing concentration of MTMOS resulted in a gradual increase in WCA values, from 64.9° for a 5% concentration up to 107.7° for 30% silane content. This can be explained by the advancing reduction of hydroxy groups in nanocellulose due to their reactivity with an increasing concentration of applied alkoxysilane [55,56,57] and due to the probable hydrogen bound interactions presented in the frame of discussion of SEM analysis (Figure 3). The addition of 30% MTMOS stabilised the WCA value over time better than 5% and 10%; after 120 s, it decreased by about 10% for M30, while for the M3 and M10 variants, the decrease was about 15–16%.

The higher hydrophobisation effect of MTMOS compared with GOPTMOS may be explained by the difference in molecular weight of the applied silanes and the number of molecules introduced into CNP films. Their molecular weights are 136.22 g/mol for MTMOS and 236.34 g/mol for GOPTMOS, and the weight concentrations of both silanes used for CNP functionalisation were the same, which means that the actual number of MTMOS molecules was about 73% higher. Even though in the GOPTMOS molecule, four functional groups can react with wood polymers compared to three in MTMOS, the number of potential reactive sites in MTMOS molecules still outnumbers that of GOPTMOS. Additionally, the lower molecular weight and size of MTMOS monomers enable their better penetrability in a nanocellulose network, resulting in easier and more effective reactivity with its hydroxy groups compared to GOPTMOS.

The addition of glycerol resulted in wettability reduction for CNPs functionalised with lower concentrations of MTMOS, while for the highest 30% silane content, no effect was observed considering the standard error values (Figure 4). Compared with GOPTMOS, MTMOS modification was less effective in surface hydrophobisation. It increased WCA values by 14.6% and 5.7% for a 5% silane concentration and by 13.2% and 23.5% for a 10% concentration at the beginning and the end of the measurement, respectively, while for GOPTMOS treatment, the observed increase in WCA values after the addition of glycerol exceeded 80% and 100%, respectively. The effect of glycerol can be explained by its ability to reduce film porosity and surface roughness, thus increasing the surface free energy and reducing wettability, as suggested by Lavrič et al. [36], while the functionalisation with hydrophobic MTMOS neutralises its hydrophilic properties, as described above for GOPTMOS-functionalised CNPs.

### 2.3. Moisture Absorption by Nanopapers

**Figure 7:**
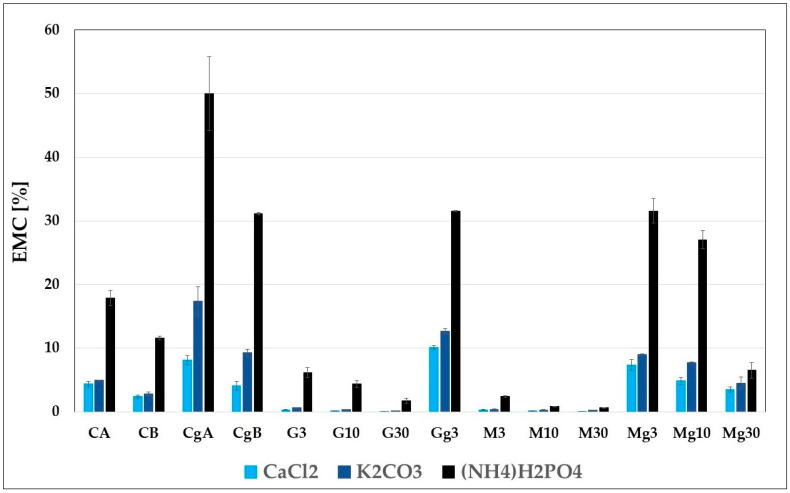
Average values of equilibrium moisture content for individual nanopapers under three different relative humidity conditions provided by saturated solutions of CaCl_2_, K_2_CO_3_ and NH_4_H_2_PO_4_; the sample abbreviation scheme is as follows: pure nanopapers CA and CB: CA—CNC:H_2_O ratio 1:1.5, CB—CNC:H_2_O ratio 1:4, modified nanopapers: XgY, where X is as follows: G—(3-Glycidyloxypropyl)trimethoxysilane and M—Methyltrimethoxysilane; g—glycerol; Y—silane content. Average values of equilibrium moisture content with standard error bars for individual nanopapers under three different relative humidity conditions provided by saturated solutions of CaCl_2_, K_2_CO_3_ and NH_4_H_2_PO_4_; the sample abbreviation scheme: XgY, where X is as follows: G—(3-Glycidyloxypropyl)trimethoxysilane and M—Methyltrimethoxysilane; g—glycerol; Y—silane concentration: 3—3%, 10—10% and 30—30%, where A—3%, B—10% and C—30%; ± standard error.

The results of equilibrium moisture content measurements under three different relative humidity conditions of 30 ± 5%, 43 ± 5%, and 93 ± 5%, provided by saturated solutions of CaCl_2_, K_2_CO_3_ and NH_4_H_2_PO_4_, respectively, are presented in Table 3. The data are also presented as a bar chart in Figure 7 to illustrate better the trends in EMC results depending on the CNP variant and RH conditions.

For all nanopaper variants, a clear correlation between RH and EMC can be seen, with EMC increasing as RH rises. Functionalisation with silanes resulted in an ascending reduction in EMC values with increasing silane concentrations in CNPs, with a higher hydrophobisation effect for MTMOS, as observed for the water contact angle measurements. The hydrophobisation effect results from the reactivity of silanes with free hydroxy groups present on nanocellulose, which limits the number of sorption sites and reduces EMC [56,58].

The addition of glycerol substantially increased the hygroscopicity of CNPs for both unmodified and silane-functionalised nanopapers. Glycerol, as a highly hygroscopic substance, apparently attracts so many water molecules that it affects the water sorption isotherm and increases the water content in nanopapers plasticised with it. Similar effects were observed for glycerol-plasticised films based on carboxymethyl cellulose, wheat starch or potato protein isolate [52,59,60], but for those based on alginate or potato starch, the effect was the opposite [61,62]. The observed differences in the effect of glycerol on the moisture content of various films plasticised with it result from the differences in water binding capacity between glycerol and individual polymers used for film preparation. When the polymer has higher hygroscopicity than glycerol, then its addition will lower the EMC of the plasticised film (as in the case of alginate or potato starch), but when the water binding capacity of a polymer is lower than that of glycerol, then plasticising such a film with glycerol increases its moisture content, thus enhancing the plasticising effect [61].

### 2.4. Resistance to Moulds

**Figure 8:**
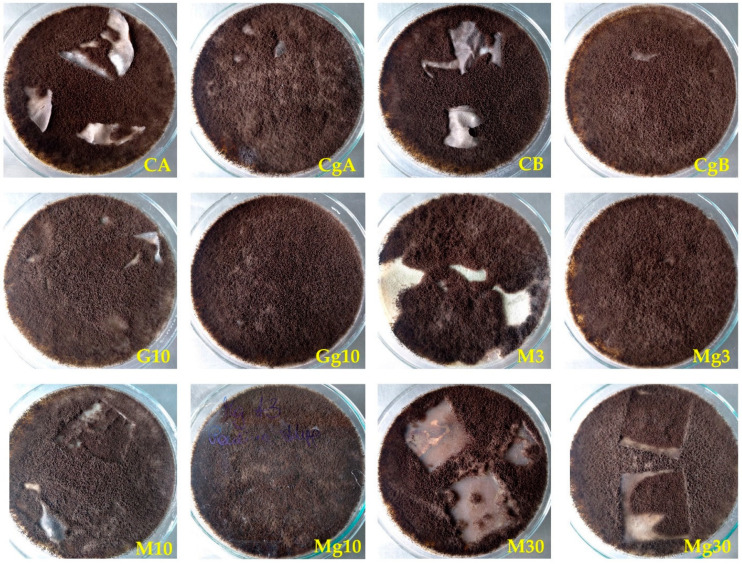
Images of nanopaper samples inoculated with a mixture of mould spores after 28 days of incubation: pure nanopapers: CgA—CNC:H_2_O ratio 1:1.5 and CgB—CNC:H_2_O ratio 1:4; nanopapers modified with (3-Glycidyloxypropyl)trimethoxysilane: G10 (10%), and the equivalent plasticised with glycerol Gg3 (3% of (3-Glycidyloxypropyl)trimethoxysilane); nanopapers modified with Methyltrimethoxysilane: M3 (3%), M10 (10%) and M30 (30%), and their equivalents plasticised with glycerol Mg3, Mg10, and Mg30, respectively.

As shown in Figure 8, none of the tested nanopapers were fully mould-resistant. Pure nanopapers (CA and CB), independently of the CNC concentration, showed partial resistance to mould growth, reaching the colonisation index of 1 after 1 week and 2.5 after 28 days of incubation. However, the addition of glycerol, although it improved their flexibility and flatness, made them more prone to fungal attack, and the plasticised pure nanopapers CgA and CgB were entirely overgrown by mould at the end of the experiment, reaching the colonisation index of 4.

It is known that selected organosilicon compounds can increase the resistance of wood and other lignocellulosic materials to fungal degradation, which is mainly achieved due to the presence of antifungal amino groups in organosilicon molecules or reduction in wood moisture content [63,64,65,66]. However, silane modifications of CNPs performed in this study were not particularly effective while tested against moulds, even though they reduced the moisture content of nanopapers. The functionalisation with (3-Glycidyloxypropyl)trimethoxysilane did not reduce CNP vulnerability to mould growth, and after one week, all treated papers, both non-plasticised and plasticised with glycerol, were overgrown by moulds, reaching the colonisation index of 4. Methyltrimethoxysilane was slightly more effective, partially increasing CNP durability against moulds. After one week, the functionalised nanopapers were covered with moulds on about 10–15% of their surfaces, and at the end of the test, they reached the colonisation index of 3.5 for 5% and 10% silane concentration and 2.5 for the highest 30% silane content. Similar none or weak antimould activity of MTMOS was observed in previous research where silane concentrations ranging from 5% to 50% were applied on wood [65,67,68]. The addition of glycerol reduced the antifungal effect of MTMOS so that the CPNs containing two lower silane concentrations were covered with mould by about 50% after 1 week and entirely overgrown after 28 days of mycological tests, while that containing 30% of MTMOS was covered with mould by about 20% after 1 week and reached the colonisation index of 3.5 at the end of the test. Therefore, from the conservation perspective, if cellulose nanopapers were to be used for wooden surface protection against mould colonisation, additional antifungal agents should be used to reduce their vulnerability to mould growth.

### 2.5. Finishing Properties of Nanopaper Surfaces

All prepared nanopapers easily stuck to the wood surface using a Vinavil Blue NPC glue. Their paintability with waterborne acrylic, oil paint, waterborne wood stain and nitro wood stain is described, scored and visualised in Table 4. A wood sample without a nanopaper on its surface was used as a reference.

As can be seen from the data in Table 4, pure nanopapers CA and CB were relatively easy to paint, with vivid colours similar to those painted on a wood sample. The colours were stable after drying except for a waterborn wood stain, which almost completely vanished during drying. That paint was generally the most troublesome for all CNPs and wood, with the colour fading or even vanishing after drying, so it seems unsuitable to paint CNPs.

Functionalisation with 5% and 10% of (3-Glycidyloxypropyl)trimethoxysilane did not change the general rating of nanopapers’ paintability but altered the colours of applied paints, making them darker when compared to pure CNPs or wood. On the other hand, the functionalisation with the highest 30% GOPTMOS content altered surface properties to such a point that the paintability of the modified G30 variant was evaluated higher (4.25 compared to 3.25 and 3.75 for unmodified variants), with colours that vanished less and were more similar to those visible on wood and unmodified CNPs.

MTMOS also affected the properties of CNPs, making the paint colours darker compared to wood and unmodified CNPs and resulting in similar paintability scores of 4.25 compared to GC, except for the 10% concentration, which for some reason worsened the effect of the waterborne wood stain and nitro wood stain, resulting in an average score of 3.25.

The effect of glycerol is highly visible on all plasticised samples. In general, glycerol improved the paintability of nanopapers so that the total score for all the plasticised samples increased compared to non-plasticised samples. The only exception is the CNP functionalised with 30% MTMOS (Mg30), for which plasticisation slightly worsened its paintability, meaning that brush marks for oil paint are more visible than on the equivalent sample without glycerol (M30). Also, for all silane functionalised samples, glycerol altered the colour of all paintings used in the experiment, making them look lighter and warmer, more similar to those painted on the wood sample, as if it neutralised the darkening effect of applied silanes.

The results of the paintability of CNPs clearly show that when used as patches protecting cracks in wood, they can be colour-matched to the surrounding wood surface by painting. However, depending on the CNP variant used, the selection of paint may be different for the wood and an individual CNP to ensure a proper adjustment of colour and shade.

## 3. Materials and Methods

### 3.1. Materials

The aqueous suspensions of cellulose nanocrystals (1.5% *w*/*w*) used for cellulose nanopaper preparation were purchased from Nano Novin Polymer Co. (Gorgan, Iran). Methyltrimethoxysilane (MTMOS) and (3-Glycidyloxypropyl)trimethoxysilane (GOPTMOS) applied for nanopaper functionalisation were purchased from Sigma Aldrich (St. Louis, MO, USA). Additionally, glycerol (Sigma Aldrich, St. Louis, MO, USA) was used as a plasticiser, and pure ethyl alcohol (96%) (Chempur, Piekary Slaskie, Poland) was used as a solvent.

### 3.2. Preparation of Pure Cellulose Nanopapers

The base suspension of CNC was mixed with distilled water in a weight ratio of 1:1.5 and 1:4. The prepared suspensions were stirred for 2 h at 8500 rpm at ambient temperature. Then, the suspensions were left for 24 h to eliminate air bubbles in the film. Simultaneously, suspensions with glycerol have been prepared in a 1:1.3 ratio. The amount of glycerol added to each suspension was calculated to achieve approximately 33% by weight for the dry film [31]. The weight ratio 1:4 was prepared to obtain the same CNC concentration in pure CNPs as in their silane functionalised versions.

Each suspension was poured onto a 6.3 cm diameter circular polypropylene pad and left to dry at ambient conditions in a sheltered place to ensure good protection against direct sunlight. After one week, nanopapers were gently detached from the pads.

### 3.3. Preparation of Functionalised Cellulose Nanopapers

Distilled water was mixed with 96% ethanol in a 1:10 ratio (*w*/*w*) and left for 24 h [6]. Then, one of the silanes, MTMOS or GOPTMOS, was added to the ethanol solution to achieve their final concentrations of 3, 10, and 30% (*w*/*w*). The pH was reduced to 4.5 using glacial acetic acid to catalyse nanocellulose functionalisation by silanes [48]. Then, the based CNC suspension was mixed with prepared solutions of silane in a 1:1 weight ratio, stirred for 2 h at ambient conditions and left for 24 h before use [6]. The six variants of functionalised nanocellulose were then diluted with distilled water in a 1:1.5 weight ratio to obtain suspensions with the desired spreadability to cover the pad evenly. Simultaneously, suspensions with glycerol have been prepared in a 1:1.3 ratio. The amount of glycerol added to each suspension was calculated to achieve approx. 33% by weight in the dry film, as for pure films [31].

The functionalised NPs were prepared by casting aqueous suspensions as described above. After one week of drying at ambient conditions, nanopapers were gently detached from the pads.

### 3.4. Evaluation of CNPs’ Selected Properties

Considering the potential future application of nanopapers to conserve wooden artefacts, their basic performance was visually assessed, including features such as ease of removal from the pads, flatness and flexibility. These properties seemed the most crucial at this research stage, and insufficient quality of nanopapers regarding these precludes their further implementation. The evaluation of listed properties was simplified by applying the 5-point scale based on visual observations and comparing individual samples with each other, where 1 represents very difficult, 2 represents difficult, 3 represents mediocre, 4 represents well, and 5 represents very well.

### 3.5. Microscopy Imaging

The cross-sectional areas of the nanopapers were examined using the stereoscopic microscope Motic SMZ-171-TLED (Motic Hong Kong Ltd., Hong Kong SAR, China). Images were taken using Motic Images Plus 2.0 software. The surface morphology and chemical composition of nanocellulose films were studied using a scanning electron microscope (SEM JEOL JEM 7001 TTLS, Tokyo, Japan) equipped with an energy dispersive X-ray (EDX) analyser using a 5 kV accelerating voltage. Before SEM imaging, the examined samples were placed on carbon tape and sputtered with a Au layer about 8 nm thick.

### 3.6. Water Contact Angle Measurements

The water contact angle (WCA) tests were intended to determine the hydrophobicity of cellulose nanopapers with both untreated and treated with silicon compounds. Nanopapers were cut into smaller pieces, approximately 2 × 2 cm, and placed on the microscope slide. The measurement of the sessile droplet contact angle was conducted using a DSA 25 goniometer from A. Krüss Optronic GmbH (Hamburg, Germany) and recorded at 20 °C and air relative humidity of 50% using a dedicated ADVANCE software. A deionised water droplet (10 μL) was placed directly on the nanopaper, and a picture of the droplet was taken within the first 5 s. After that, the pictures were taken every 30 s until 120 s. WCA was calculated for each CNP sample by averaging the values measured from the left and right sight of the water droplet; three replicates of each variant were measured.

### 3.7. Moisture Absorption Measurements

To evaluate moisture absorption by pure and modified nanopapers, they were cut into smaller pieces of approx. 2 cm × 2 cm, oven-dried at 60 °C to a constant weight, weighted, and then placed in hermetic chambers above aqueous oversaturated solutions of CaCl_2_, K_2_CO_3_ and NH_4_H_2_PO_4_, which provided an air relative humidity of 30 ± 5%, 43 ± 5%, and 93 ± 5%, respectively. The samples were stored in the containers at a temperature of 23 ± 2 °C until they reached the equilibrium moisture content (EMC) (i.e., the mass change of the sample within 24 h was less than 1%) and then weighed again. Three replicates of each variant were measured. For each relative humidity, EMC (%) was calculated as follows:

$$EMC=\left(\frac{m_{1}-m_{0}}{m_{1}}\right)\times 100\%$$

where EMC is the equilibrium moisture content, m_0_ is the mass of a dry sample before the measurement, and m_1_ is the mass of a wet sample at the end of the measurement.

### 3.8. Resistance to Mould

The mycological test was performed to determine the level of nanopaper colonisation by moulds based on the ASTM D5590 standard [69]. The study was conducted on all samples to determine whether and to what extent the addition of silanes and glycerine affects fungal infestation. In the experiment, a mixture of the following fungi cultures was applied: *Aspergillus niger* van Tieghem ATCC 6275, *Trichoderma viride* Pers. ex Fr. ATCC 36316, *Paecilomyces variotii* Bainer ATCC 26820, *Chaetomium globosum* Kunze ex Fr. ATCC 6205 and *Penicillium funiculosum* Thom ATCC 11979. All nanopaper samples were sterilised in a steam atmosphere in an autoclave (121 °C, 10 bars, 20 min). Then, each sample was placed on a 3–4 nm thick malt agar medium layer (20 g of agar, 30 g of malt extract, 4 g/L Czapek-Dox mineral mixture per 1 L of water) in a separate Petri dish and inoculated with a water suspension of a fungal spore mixture; 5 replicates of each type were examined. The inoculated nanopapers were incubated in the cultivation chamber at a constant temperature of 28 ± 1 °C and humidity of 80 ± 5% for 28 days. The growth of fungi was monitored, and after 28 days of incubation, the colonisation index was determined based on the scale presented in Table 5.

### 3.9. Adherence to the Wood Surface and Finishing Properties of Nanopaper Surfaces

The manufactured nanopapers were intended to serve as covers for gaps and cracks in wooden artefacts. Therefore, their adherence to the wood surface was examined by glueing the nanopaper with a polyvinyl acetate glue named Vinavil Blue NPC (CTS, Briosco, Italy) to the surface of a wooden block (*Populus tremula* L.) with dimensions 2 cm × 2 cm × 2 cm, with a specially prepared V-shaped hole that imitated a natural gap in a wooden object. Additionally, it was possible to alter their appearance and make them similar to the surrounding surface of the conserved wooden object by painting them with selected paints, such as waterborne acrylic (Jiangsu Phoenix Art Materials Technology Co., Ltd., Jiangsu, China; colour: burnt umber), oil paint (Renesans, Kobylnica, Poland; colour: burnt umber), waterborne wood stain (Sopur, Bydgoszcz, Poland, colour: brown (Brunat)) and nitro wood stain (Sopur, Bydgoszcz, Poland, colour: brown (Brunat)).

## 4. Conclusions

In this study, cellulose nanopapers intended to serve as patches to protect degraded, fissured surfaces of wooden artefacts against water, dust, and dirt penetration were produced from aqueous suspensions of cellulose nanocrystals. The effects of glycerol and silane modification ((3-Glycidyloxypropyl)trimethoxysilane and Methyltrimethoxysilane) on CNP properties were investigated.

The obtained pure CNC nanopapers were very brittle, stiff, wavy and undulating, with relatively homogeneous and compact structures. Functionalisation with silanes made the CNP structures more packed, with slightly smoother surfaces. It did not affect (lower concentrations) or improve the flexibility and flatness of the CNPs (higher concentrations), with GOPTMOS having a higher impact compared to MTMOS. Glycerol addition had a plasticising effect on pure and modified CNPs, increasing their flexibility and resistance to fracture and making them more flat and less puckered. Unfortunately, when glycerol was combined with 10% and 30% of GOPTMOS, it turned the CNC suspension into a dense, sticky, semi-liquid substance that did not form a film even after drying for two weeks.

The structural and chemical changes in CNPs imparted by glycerol and silanes translate into the moisture properties of CNPs. Functionalisation with silanes hydrophobises nanopapers, while adding hygroscopic glycerol increases the EMC values of both pure and silane-functionalised nanopapers, which enhances its plasticising effect, but at the same time, it increases the WCA values for their surfaces, which may seem contradictory. The reduction in the surface porosity of pure CNPs by glycerol has a more pronounced effect on its wettability than the hygroscopic nature of glycerol molecules. In the case of CNPs functionalised with silanes, we hypothesise that the reactivity of silane molecules with -OH groups of glycerol neutralises its hygroscopic nature, further contributing to the reduction in CNP surface wettability.

Although nanopaper functionalisation with selected silanes reduces the hygroscopicity of the material, it is insufficient to provide them with resistance to moulds. The increased hygroscopicity of CNPs plasticised by glycerol makes them more prone to colonisation by mould.

All the prepared nanopapers glued to wooden blocks hold onto their surface well and can be colour-matched using different paints. Glycerol slightly improves the paintability of CNPs, presumably due to the smoothening effect on their surfaces. Since functionalisation with silanes alters the colour of paint applied on CNPs compared to that obtained on wood, selecting a painting shade should be carried out individually to ensure a proper adjustment between the wood and the CNP surface.

From the conservation perspective, the most suitable nanopapers were those treated with 30% GOPTMOS (G30) and with glycerol and 3% GOPTMOS (Gg3), and they are worth further study and improvements. They were flexible and flat, with good paintability. However, the latter was highly hygroscopic, while the former was hydrophilic, which needs improvement. Both variants were prone to fungal attack; hence, additional protection would be needed if they were supposed to provide some resistance to moulds. On the other hand, the use of a different plasticiser, which would introduce better flexibility to nanopapers, could make the variants treated with 30% MTMOS (Mg30, M30) worth further research since they were hydrophobic, provided some resistance to moulds and had good paintability, which is important from the practical perspective.

In summary, cellulose nanopapers seem to be a promising solution for protecting the cracked surface of wooden artefacts against further degradation due to external conditions. However, to provide them with properties suitable from the conservation perspective, further plasticisation and modification of CNC are necessary. Glycerol serves well as a plasticiser, improving the structural and mechanical properties of CNP patches. However, its hygroscopic nature undesirably increases the hygroscopicity of CNPs, making them more prone to fungal infestation and presumably dimensional changes, which may alter their protective function. Therefore, a less hygroscopic plasticiser may be more appropriate for such purposes. Functionalisation with silanes improves the mechanical and moisture properties of CNPs, which justifies their application for such solutions.

## Figures and Tables

**Table 1:** The composition and selected properties of pure CNP evaluated according to a 5-point visual scale, where 1 means very difficult, 2 is difficult, 3 is mediocre, 4 is well, and 5 is very well; the abbreviation scheme is as follows: CgY, where C is cellulose nanocrystals, g is glycerol and Y is CNC to H_2_O ratio, where A is 1:1.5 and B is 1:4.

Sample ID	Glycerol	CNC:H_2_O Ratio (*w*/*w*; Wet State)	General Properties			
			Flexibility	Flatness	Ease of Removal from the Pad	Average Score
CA	-	1:1.5	1	2	5	2.7
CgA	+	1:1.5	4	4	5	4.3
CB	-	1:4	1	2	5	2.7
CgB	+	1:4	4	4	5	4.3

**Table 2:** The composition and selected properties of modified CNP evaluated according to a 5-point visual scale, where 1 means very difficult, 2 is difficult, 3 is mediocre, 4 is well, and 5 is very well; the abbreviation scheme is as follows: XgY, where X is as follows: G—nanocellulose functionalised with (3-Glycidyloxypropyl)trimethoxysilane and M—nanocellulose functionalised with Methyltrimethoxysilane; g—glycerol; Y–silane concentration: 3—3%, 10—10% and 30—30%.

Sample ID	Silane Concentration	Glycerol	Modified CNC:H_2_O Ratio (*w*/*w*; Wet State)	General Properties			
				Flexibility	Flatness	Ease of Removal from the Pad	Average Score
G3	3%	-	1:1.5	2	4	4	3.3
Gg3	3%	+	1:1.5	5	5	2	4.0
G10	10%	-	1:1.5	4	4	4	4.0
Gg10	10%	+	1:1.5	-	-	1	-
G30	30%	-	1:1.5	5	5	2	4.0
Gg30	30%	+	1:1.5	-	-	1	-
M3	3%	-	1:1.5	1	2	4	2.3
Mg3	3%	+	1:1.5	4	4	2	3.3
M10	10%	-	1:1.5	1	4	3	2.7
Mg10	10%	+	1:1.5	3	3	4	3.3
M30	30%	-	1:1.5	4	4	4	4.0
Mg30	30%	+	1:1.5	5	5	4	4.7

**Table 3:** Average values of equilibrium moisture content for individual nanopapers under three different relative humidity conditions provided by saturated solutions of CaCl_2_, K_2_CO_3_ and NH_4_H_2_PO_4_; the sample abbreviation scheme is as follows: pure nanopapers CA and CB: CA—CNC:H_2_O ratio 1:1.5 and CB—CNC:H_2_O ratio 1:4; modified nanopapers: XgY, where X is as follows: G—(3-Glycidyloxypropyl)trimethoxysilane and M—Methyltrimethoxysilane; g—glycerol; Y—silane concentration, where A—3%, B—10% and C—30%; ± standard error.

Sample ID	EMC [%]		
	CaCl_2_ (RH ≈ 30 ± 5%)	K_2_CO_3_ (RH ≈ 43 ± 5%)	NH_4_H_2_PO_4_ (RH ≈ 93 ± 5%)
CA	4.38 ± 0.47	4.97 ± 0.05	17.90 ± 1.21
CB	2.41 ± 0.21	2.84 ± 0.31	11.55 ± 0.30
CgA	8.16 ± 0.67	17.34 ± 2.34	50.01 ± 5.81
CgB	4.11 ± 0.72	9.35 ± 0.46	31.18 ± 0.21
G3	0.31 ± 0.07	0.70 ± 0.00	6.17 ± 0.81
G10	0.16 ± 0.01	0.39 ± 0.02	4.41 ± 0.54
G30	0.08 ± 0.02	0.16 ± 0.02	1.78 ± 0.38
Gg3	10.13 ± 0.33	12.70 ± 0.40	31.57 ± 0.09
M3	0.30 ± 0.09	0.43 ± 0.03	2.44 ± 0.15
M10	0.21 ± 0.02	0.32 ± 0.05	0.83 ± 0.00
M30	0.11 ± 0.01	0.26 ± 0.05	0.70 ± 0.00
Mg3	7.40 ± 0.87	9.06 ± 0.07	31.58 ± 1.90
Mg10	4.88 ± 0.54	7.73 ± 0.10	27.07 ± 1.40
Mg30	3.57 ± 0.40	4.56 ± 0.96	6.53 ± 1.23

**Table 4:** The paintability of pure and functionalised CNPs with a waterborne wood stain (1), nitro wood stain (2), oil paint (3) and waterborne acrylic paint (4). Evaluation of paintability was made according to a 5-point scale, where 1 means difficult, 2 means mediocre, 3 means well, 4 means quite well and 5 means very well. ID—sample type abbreviation, including pure nanopapers CA—CNC:H_2_O ratio 1:1.5, CB—CNC:H_2_O ratio 1:4 and modified nanopapers. XgY, where X is as follows: G—(3-Glycidyloxypropyl)trimethoxysilane and M—Methyltrimethoxysilane; g—glycerol; Y—silane concentration: 3—3%, 10—10% and 30—30%; W—pine wood sample for a reference.

ID	Paintability of Nanopapers				
	Waterborne Wood Stain (1)	Nitro Wood Stain (2)	Oil Paint (3)	Waterborne Acrylic Paint (4)	Average Score
CA	Score: 2 Spreads easily over the surface Deeply penetrates the surface Weak surface colouring Colour vanishes after drying	Score: 4 Does not spread over the surface Easily applied to the surface Good surface colouring Colour remains after drying	Score: 3 Quite difficult to spread over the surface Visible brush marks after drying Good surface colouring Colour remains after drying	Score: 4 Spreads well on the surface Visible brush marks after drying Good surface colouring Colour remains after drying	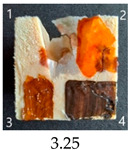
CB	Score: 2 Spreads easily over the surface Deeply penetrates the surface Weak surface colouring Colour vanishes after drying	Score: 4 Does not spread over the surface Easily applied to the surface Good surface colouring Colour remains after drying	Score: 5 Spreads well on the surface Visible brush marks after drying Great surface colouring Colour remains after drying	Score: 4 Spreads well on the surface Visible brush marks after drying Good surface colouring Colour remains after drying	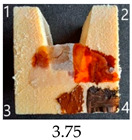
CgA	Score: 4 Does not spread over the surface Slightly penetrates the surface Good surface colouring Colour fades after drying	Score: 5 Does not spread over the surface Easily applied to the surface Great surface colouring Colour remains after drying	Score: 5 Spreads well on the surface Visible brush marks after drying Great surface colouring Colour remains after drying	Score: 5 Spreads well on the surface Visible brush marks after drying Great surface colouring Colour remains after drying	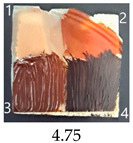
CgB	Score: 4 Does not spread over the surface Slightly penetrates the surface Good surface colouring Colour fades after drying	Score: 5 Does not spread over the surface Easily applied to the surface Great surface colouring Colour remains after drying	Score: 5 Distributes well on the surface Slightly visible brush marks after drying Great surface colouring Colour remains after drying	Score: 5 Distributes well on the surface Visible brush marks after drying Great surface colouring Colour remains after drying	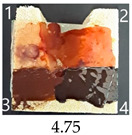
G3	Score: 2 Spreads quickly over the surface Deeply penetrates the surface Weak surface colouring Colour vanishes after drying	Score: 4 Does not spread over the surface Easily applied to the surface Good surface colouring Colour remains after drying	Score: 3 Quite difficult to spread on the surface Visible brush marks after drying Good surface colouring Colour remains after drying	Score: 5 Distributes well on the surface Visible brush marks after drying Great surface colouring Colour remains after drying	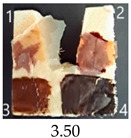
G10	Score: 2 Spreads quickly over the surface Deeply penetrates the surface Weak surface colouring Colour vanishes after drying	Score: 4 Does not spread over the surface Easily applied to the surface Good surface colouring Colour remains after drying	Score: 3 Quite difficult to spread on the surface Visible brush marks after drying Medium surface colouring Colour remains after drying	Score: 4 Spreads well on the surface Visible brush marks after drying Good surface colouring Colour remains after drying	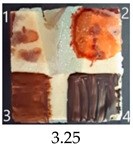
G30	Score: 3 Spreads quickly over the surface Slightly penetrates the surface Quite good surface colouring Colour remains after drying	Score: 5 Does not spread over the surface Easily applied to the surface Great surface colouring Colour remains after drying	Score: 5 Spreads well on the surface Slightly visible brush marks after drying Great surface colouring Colour remains after drying	Score: 4 Spreads well on the surface Visible brush marks after drying Great surface colouring Colour remains after drying	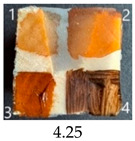
Gg3	Score: 4 Does not spread over the surface Slightly penetrates the surface Good surface colouring Colour fades after drying	Score: 4 Does not spread over the surface Easily applied to the surface Good surface colouring Colour fades after drying	Score: 4 Spreads well on the surface Visible brush marks after drying Great surface colouring Colour remains after drying	Score: 5 Spreads well on the surface Slightly visible brush marks after drying Great surface colouring Colour remains after drying	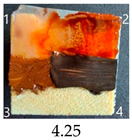
M3	Score: 3 Spreads over the surface Slightly penetrates the surface Quite good surface colouring Colour fades after drying	Score: 4 Does not spread over the surface Easily applied to the surface Good surface colouring Colour remains after drying	Score: 5 Spreads well on the surface Slightly visible brush marks after drying Great surface colouring Colour remains after drying	Score: 5 Spreads well on the surface Slightly visible brush marks after drying Great surface colouring Colour remains after drying	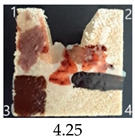
M10	Score: 2 Spreads over the surface Penetrates the surface Poor surface colouring Colour vanishes after drying	Score: 3 Spreads over the surface Easily applied to the surface Poor colouring Colour remains after drying	Score: 4 Spreads well on the surface Visible brush marks after drying Great surface colouring Colour remains after drying	Score: 4 Spreads well on the surface Visible brush marks after drying Great surface colouring Colour remains after drying	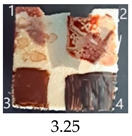
M30	Score: 3 Spreads over the surface Slightly penetrates the surface Quite good surface colouring Colour fades after drying	Score: 5 Does not spread over the surface Easily applied to the surface Very good surface colouring Colour remains after drying	Score: 5 Spreads well on the surface Slightly visible brush marks after drying Great surface colouring Colour remains after drying	Score: 4 Spreads well on the surface Visible brush marks after drying Great surface colouring Colour remains after drying	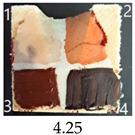
Mg3	Score: 4 Does not spread over the surface Slightly penetrates the surface Good surface colouring Colour fades after drying	Score: 5 Does not spread over the surface Easily applied to the surface Great surface colouring Colour remains after drying	Score: 5 Spreads well on the surface Slightly visible brush marks after drying Great surface colouring Colour remains after drying	Score: 5 sPreads well on the surface Slightly visible brush marks after drying Great surface colouring Colour remains after drying	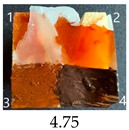
Mg10	Score: 4 Does not spread over the surface Slightly penetrates the surface Good surface colouring Colour remains after drying	Score: 4 Does not spread over the surface Slightly penetrates the surface Good surface colouring Colour remains after drying	Score: 5 Spreads well on the surface Slightly visible brush marks after drying Great surface colouring Colour remains after drying	Score: 4 Spreads well on the surface Visible brush marks after drying Great surface colouring Colour remains after drying	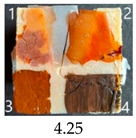
Mg30	Score: 3 Spreads over the surface Slightly penetrates the surface Quite good surface colouring Colour fades after drying	Score: 5 Does not spread over the surface Easily applied to the surface Very good surface colouring Colour remains after drying	Score: 4 Spreads well on the surface Visible brush marks after drying Very good surface colouring Colour remains after drying	Score: 4 Spreads well on the surface Visible brush marks after drying Very good surface colouring Colour remains after drying	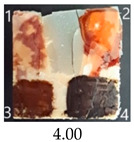
W	Score: 4 Does not spread over the surface Deeply penetrates the surface Quite good surface colouring Colour fades a bit after drying	Score: 5 Does not spread over the surface Easily applied to the surface Very good surface colouring Colour remains after drying	Score: 5 Spreads well on the surface Slightly visible brush marks after drying Very good surface colouring Colour remains after drying	Score: 5 Spreads well on the surface Slightly visible brush marks after drying Very good surface colouring Colour remains after drying	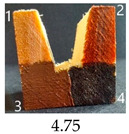

**Table 5:** The scale of the CNP surface colonisation by moulds according to ASTM D5590 with a corresponding colonisation index based on visual assessment.

Index	Description
0z	No colonisation of the CNP surface by fungi, visible zone of inhibition
0	No colonisation of the CNP surface by fungi
1	1–10% of the CNP surface colonised by fungi
2	11–30% of the CNP surface colonised by fungi
3	31–60% of the CNP surface colonised by fungi
4	More than 60% of the CNP surface colonised by fungi

## Data Availability

The data that support the findings of this study are available from the corresponding author, [M.B.], upon reasonable request.
